# Supplementary material for: Variation in the Floral Scent Chemistry of Nymphaea ‘Eldorado’, a Valuable Water Lily, with Different Flowering Stages and Flower Parts
Source: Plants (Basel). 2024 Mar 24;13(7):939. doi: 10.3390/plants13070939 (PMC11013332; doi:10.3390/plants13070939)
Supplement: Supplementary file 1 [file plants-13-00939-s001.zip › plants-2885832-supplementary.pdf]

Supplementary Figures:

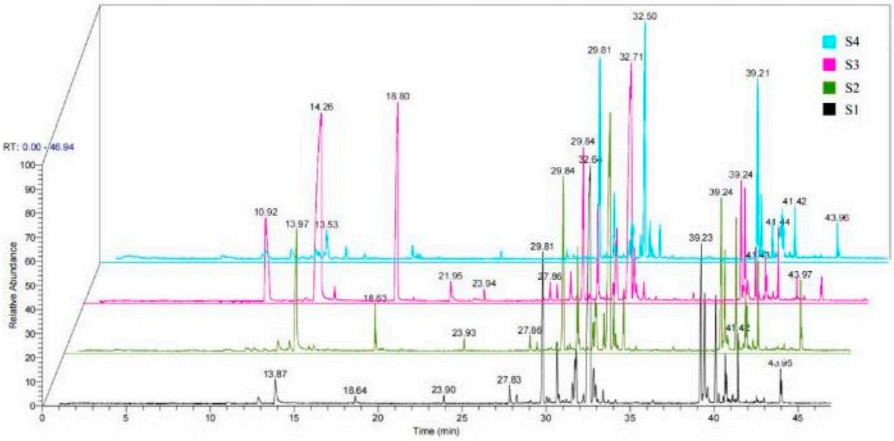

Figure S1. The GC-MS total ion chromatograms (TICs) for the volatile chemical profiles of *N*. 'Eidorado' at different flowering stages.

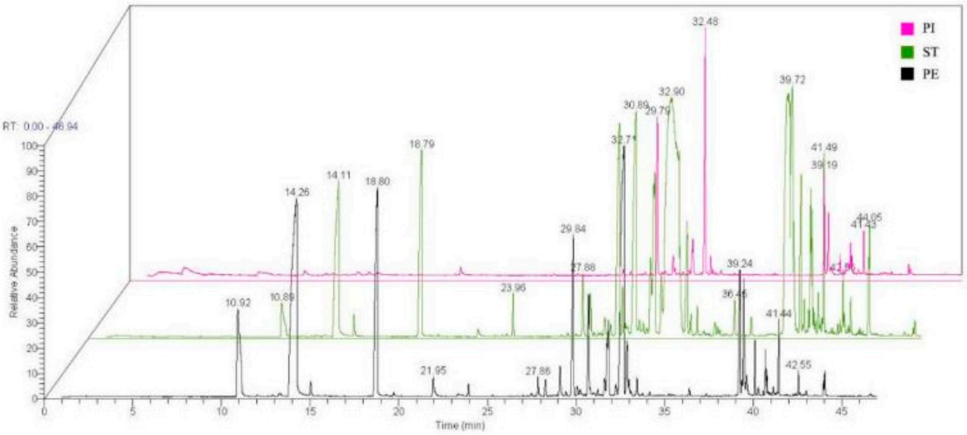

Figure S2. The GC-MS total ion chromatograms (TICs) for the volatile chemical profiles of different parts of *N*. 'Eidorado' flower.
